# Influence of Environmental Factors on the Active Substance Production and Antioxidant Activity in *Potentilla fruticosa* L. and Its Quality Assessment

**DOI:** 10.1038/srep28591

**Published:** 2016-07-04

**Authors:** Wei Liu, Dongxue Yin, Na Li, Xiaogai Hou, Dongmei Wang, Dengwu Li, Jianjun Liu

**Affiliations:** 1 Agricultural College, Henan University of Science and Technology, Luoyang 471003, China; 2 College of Agricultural Engineering, Henan University of Science and Technology, Luoyang 471003, China; 3 College of Forestry, Northwest A&F University, Yangling 712100, Shaanxi, China; 4 College of Landscape Architecture and Arts, Northwest A&F University, Yangling 712100, Shaanxi, China

## Abstract

Environmental factors may influence types and contents of active substances. This study investigated the influence of environmental factors on the active substance contents and antioxidant activity of *Potentilla fruticosa* L. from different regions of China. Also, HPLC fingerprint similarity analysis (SA) coupled with hierarchical cluster analysis (HCA) and discriminant analysis (DA) were further introduced for the accurate classification and quality assessment of *P. fruticosa*. The results showed that altitude was significantly and negatively correlated to the content of tannin (*P* < 0.05). Annual sunshine duration and altitude were significantly and positively correlated to the flavonoids content, rutin content and antioxidant activity (*P* < 0.05). Annual mean temperature was significantly and negatively correlated to the content of total phenolics, while altitude was significantly and positively correlated to the content of total phenolics (*P* < 0.05). Eight samples were unambiguously separated into three groups. Two types of discriminant functions with a 100% discrimination ratio were constructed. All data consistently supported the conclusion that *P. fruticosa* produced from Kangding, Sichuan Province had high quality among all samples, therefore, Kangding in Sichuan Province with favorable environmental conditions is recommended as a preferable production location.

The quality of traditional medicine, which plays a very important role in the health system of China, is determined by its active substances produced by the plants. *Potentilla* species have been used for a long time in traditional medicine for its curative properties. In Chinese traditional medicine *Potentilla* extracts have been used to treat diarrhoea, hepatitis, rheuma and scabies and as a remedy for detoxification^1^,^2^. *Potentilla fruticosa* L.is a species of hardy deciduous flowering shrub in the *Potentilla* genus of the family *Rosaceae*, native to the cool temperate and subarctic regions of the northern hemisphere, often growing at high altitudes in mountains^3^. Apart from common application as a garden plant, it also has numerous medicinal virtues^4^. Extracts of *P. fruticosa* have been shown to possess relatively high concentrations of phenolic acids and flavonoids and powerful radical scavenging capacity^5^,^6^,^7^. The activity of some extracts was higher than that of the synthetic antioxidant BHT (butylated hydroxytoluene) and of extracts isolated from *Salvia officinalis* L., which contains powerful antioxidants^7^. Moreover the leaves of *P. fruticosa* have applications as food additives and an ingredient in cosmetic products^8^. Similarly the same or other (local) *Potentilla* species have been used in traditional medicine of different cultures in Asia, Europe and Northern America. Modern scientific researches have confirmed that the material foundations of traditional Chinese herbal drugs are the different chemical constituents (most of them are secondary metabolites) that are contained in different raw plant materials^9^.

In China, *P. fruticosa* is commonly known as the “Jinlaomei medicine” and “Gesanghua” and is widely distributed in Qinghai, Gansu, Sichuan, Yunnan, Tibet and Heilongjiang, its altitude ranges from 400 to 5000 m^10^,^11^. For this widespread species, active substances that are contained in the same plant species may be different in types, contents, and proportions of the constituents because of the environmental differences in growing locations. Active substances are the result of the interaction between plants and the environment in the long evolution process, and its production and changes have a strong correlation and association with the environment^12^. Certain substances are only synthesized under specific environments, or the contents of certain substances may significantly increase under specific environments^12^. Studies have reported on the influences of growth environment on active substances of other medicinal plants. For example, altitude and annual mean temperature were significantly and positively correlated to the contents of chlorogenic acid and flavonoids (*P* < 0.05); annual sunshine duration was significantly and positively correlated to the content of geniposidic acid (*P* < 0.05), while annual mean temperature was significantly and negatively correlated to the content of geniposidic acid (*P* < 0.05) in *Eucommia ulmoides* Oliv^9^. In *Betula pendula* Roth., altitude was positively correlated to the contents of flavonoids^13^. Among the *Sinopodophyllum hexandrum* (Royle) T.S. Ying populations, the existing variations in podophyllotoxin content were proved to be coupled with geographical altitude and local ecological conditions (temperature, rainfall, humidity, soil pH, etc.) but not with genetic basis^14^,^15^. Previous studies have demonstrated that medicinal plants that grow in various environments produce different active substance contents because of their wide distribution in different geological zones. This will result in variations in their internal qualities in the same species from different growing regions^9^, making the quality assessment of widespread species *P. fruticosa* extremely crucial. Additionally, because applications for *P. fruticosa* are growing consistently, exploring a reliable quality assessment method is essential. Traditionally, the qualities of traditional Chinese herb medicines (TCHMs) were assessed by their external appearance, and by the experiences of medicinal practice of ancient physicians^16^. In this process, the concept of so called geo-authentic herbal drugs was established. It is assumed that most geo-authentic traditional herbs produced in their native geographical area contain adequate effective chemical constituents. For example, only *Picrorhiza scrophulariiflora* Pennell., one of the well-known herbal drugs in TCHM, produced in Tibet, China is officially recognized for use in medicinal practice^17^. By contrast, only *Panax ginseng* C. A. Mey., produced in northeastern China is officially recognized as medicinal drug^17^. Currently, high performance liquid chromatography (HPLC) techniques coupled with multivariate statistical methods (chemometric methods) have been employed extensively to classify and distinguish various herbs^18^,^19^,^20^, which is regarded as a reasonable approach for the quality evaluation of complicated TCHMs^21^. For example, twelve raw herbs of *Artemisia selengensis* Turcz ex Besser collected from five provinces of China were better clustered into three groups by HPLC combined with chemometrics methods viz. similarity analysis (SA), hierarchical clustering analysis (HCA) and principal component analysis (PCA); with fingerprint matching and discrimination, classification of samples becomes the preferred method of sample comparison for quality assurance requirements^22^. HPLC fingerprint analysis, PCA, and cluster analysis (CA) were introduced for quality assessment of Cortex cinnamomi and showed that 30 samples of Cortex cinnamomi from different species and geographic locations were rationally divided into three groups^23^. Chemical fingerprints of Lingzhi (*Ganoderma*) strains were evaluated statistically using HCA and discriminant analysis (DA) in order to classify the samples and to identify key categorizing parameters. The results indicated fifteen representative Lingzhi strains were separated into three groups, thereby confirming divisions based on morphological characteristics and providing reference data for its quality assessment^20^. Therefore, in order to produce qualified TCHMs, the influences of ecological factors should be investigated to determine the suitable production locations. On the other hand, quality evaluation of TCHMs should be performed by the HPLC fingerprint method coupled with multivariate statistical techniques.

In this study, the authors therefore investigated the influences of environmental factors on the main active substances (tannin, total flavonoids, rutin, total phenolics) and antioxidant activity of *P. fruticosa* from representative growing locations throughout China where *P. fruticosa* has been naturally growth according to historical records. Moreover, HPLC fingerprint similarity analysis (SA) coupled with hierarchical cluster analysis (HCA) and discrimination analysis (DA) were further introduced for accurate identification, classification and quality assessment of *P. fruticosa*. The present study aims at (1) clarifying the environmental factors affecting the production of active ingredients and antioxidant activity of *P. fruticosa*, (2) classifying the same *P. fruticosa* species from different regions based on HPLC fingerprint data for quality assessment, (3) suggestting the best production areas for this wild species, and (4) promoting its reasonable exploitation for the raw material production of medicinal drugs rather than arbitrary harvesting wild resources.

## Results

### Validation of the HPLC procedure

**Figure 1:**
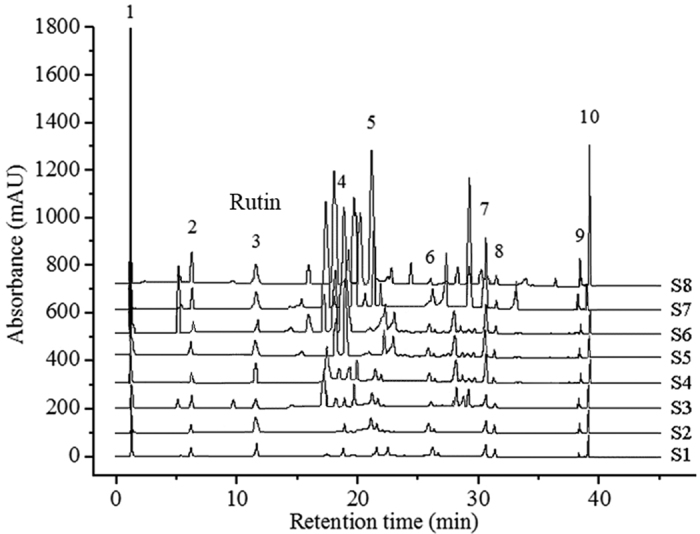
Chromatography of all the samples from eight different growing locations. S1, Mei county, Shaanxi; S2, Diebu, Gansu; S3, Huzhu, Qinghai; S4, Jingyuan, Ningxia; S5, Yongdeng, Gansu; S6, Shangri-la, Yunnan; S7, Ningchi, Tibet; S8, Kangding, Sichuan.

The precision and repeatability of the method were assessed using seven injections of sample solutions and six replicates of dry solid plant samples (re-extracting), respectively^23^. For the common peaks (Fig. 1, peaks 1–10) from duplicate injections, the relative standard deviations (RSD) of relative retention times (RRT) and relative peak areas (RPA) were found to be in the range of 0.02–0.06% and 0.22–2.91%, respectively (n = 7), which were calculated, respectively, to be 0.03–0.13% and 1.29–2.74% for six replicates of the solid samples (n = 6). To evaluate the accuracy of the method, we conducted a recovery experiment in which quantified analytes were mixed with specific amounts of standard components. The average percent recoveries for common peaks were in the range of 97.24 ± 0.02% to 104.31 ± 0.04%. The RSDs shifted from 1.19% to 2.31% (n = 6). The limit of detection (LOD) (signal/noise = 3) and the limit of quantification (LOQ) (signal/noise = 10) of the ten compounds (ten common peaks) varied within the range 1.95–3.12 ng/mL and 7.82–10.36 ng/mL, respectively. The stability of common peaks in the sample solutions that were maintained for 0–24 h was evaluated by determining their RPAs. The RSDs of RRTs and RPAs were found to be less than 3%. These results demonstrated that the conditions for the fingerprint analysis were optimal.

### Differences in active ingredient contents

**Figure 2:**
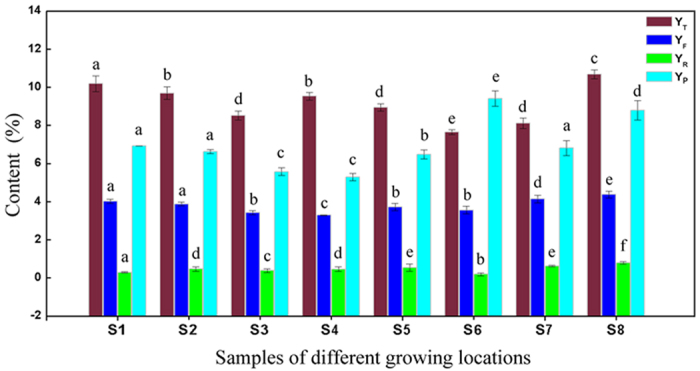
Differences of active ingredient contents in the leaves of *P. fruticosa* in different growing locations. For the same variable, bars with no letters in common are significantly different (*P* < 0.05). Y_T_ (%), tannin content; Y_F_ (%), total flavonoids content; Y_R_ (%), rutin content; Y_P_ (%), total phenolics content. S1, Mei county, Shaanxi; S2, Diebu, Gansu; S3, Huzhu, Qinghai; S4, Jingyuan, Ningxia; S5, Yongdeng, Gansu; S6, Shangri-la, Yunnan; S7, Ningchi, Tibet; S8, Kangding, Sichuan.

The active ingredient contents of *P. fruticosa* leaves differed significantly because of their various growing locations (*P* < 0.05, Fig. 2). The highest tannin content (10.68%), flavonoids content (4.37%), and rutin content (0.79%) were found in leaves from the Kangding, Sichuan (S8), whereas the lowest tannin content (7.64%) and rutin content (0.19%) were observed in leaves from Shangri-la, Yunnan (S6). The lowest flavonoids content (3.29%) was found in leaves from Jingyuan, Ningxia (S4). Moreover, the leaves from Shangri-la, Yunnan (S6) had higher phenolic contents than any other growing location, with total phenolic contents at 9.40%. By contrast, leaves from the Jingyuan, Ningxia (S4) had the lowest total phenolic contents (5.29%). These rich differences may be due to ecological factors, genetic factors and the status of secondary metabolism in different growing locations.

### DPPH radical scavenging activity

**Figure 3:**
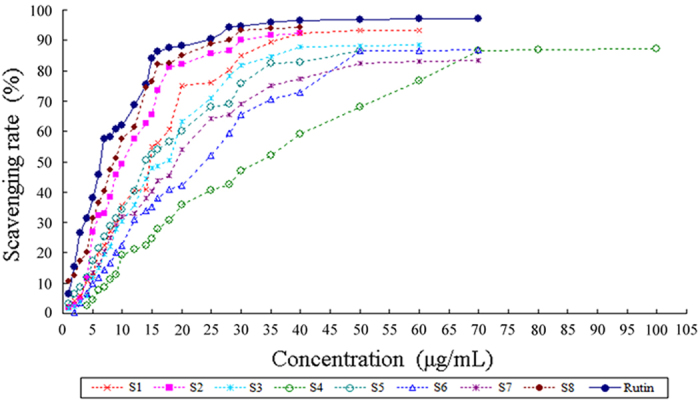
DPPH radical scavenging activity of eight *P. fruticosa* samples from different growing locations.

For evaluation of antioxidant activity of *P. fruticosa*, DPPH radical scavenging activity^24^ of different samples were compared and showed in Fig. 3. The scavenging effects of different locations increased with concentrations between 1 and 100 μg/mL. Among all samples, the highest radical scavenging activity was obtained for *P. fruticosa* collected from S8 (Kangding, Sichuan) with the lowest average IC_50_ value of 9.24 ± 0.04 μg/ml, followed by S2 (Diebu, Gansu, 10.01 ± 0.06 μg/ml). For *P. fruticosa* samples from eight regions, the IC_50_ values changed with the order: Rutin (5.85 ± 0.02 μg/ml) < S8 (9.24 ± 0.04 μg/ml) < S2 (10.01 ± 0.06 μg/ml) < S5 (14.25 ± 0.11 μg/ml) < S1 (14.76 ± 0.21 μg/ml) < S3 (17.63 ± 0.37 μg/ml) < S7 (18.94 ± 1.04 μg/ml) < S6 (23.68 ± 1.28 μg/ml) < S4 (32.35 ± 2.65 μg/ml). Compared with rutin standard, there was no significant difference (*P* < 0.05) on the antioxidant activities of S8, implying that S8 could have same scavenging effect with rutin standard at adequate concentration. These data indicated that *P. fruticosa* had high antioxidant activity, and the antioxidant activity of the same *P. fruticosa* species of different locations varied immensely from region to region.

### Ferric reducing activity power (FRAP) assay

**Figure 4:**
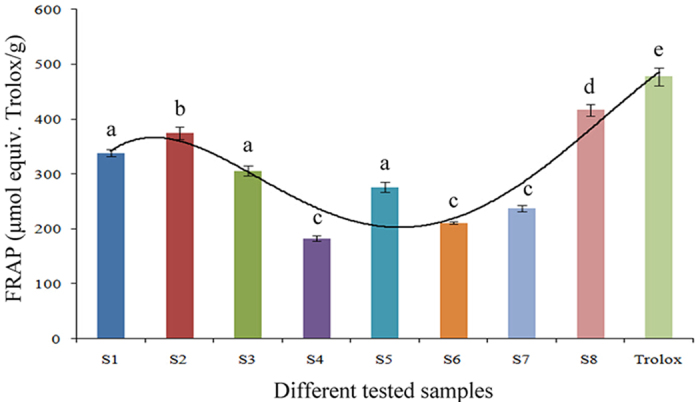
Reducing power of eight *P. fruticosa* samples from different growing locations for ferric reducing activity power (FRAP) assay.

The antioxidant properties of the samples from different growing locations were evaluated based on their reducing ability using the FRAP assay. The FRAP values obtained from eight samples were significantly different (*p* < 0.05), which presented an inverted parabola (Fig. 4). In details, as to *P. fruticosa* derived from eight regions, the FRAP value ranged from 195.58 ± 6.86 to 416.58 ± 8.62 μmol equiv. Trolox/g. *P. fruticosa* sampled from S8 (Kangding, Sichuan) possessed the highest antioxidant capacity with a FRAP value value of 416.58 ± 8.62 μmol equiv. Trolox/g, followed by samples from S2 (Diebu, Gansu) and S1 (Mei county, Shaanxi) with values of 374.43 ± 6.47 and 337.91 ± 3.41 μmol equiv. Trolox/g, respectively.

The FRAP values showed the same order of activity observed in the DPPH method: S8 > S2 > S1 > S3 > S5 > S7 > S6 > S4. Based on these results, we concluded that *P. fruticosa* samples presented not only free radical scavenge capacity but also reducing capacity. Also, *P. fruticosa* samples from Kangding, Sichuan (S8) was strongest in antioxidant capacity among all samples, which was close to the rutin and trolox standard. Furthermore, the antioxidant capacity of *P. fruticosa* samples exhibited significant differences in the same species from different regions (*p* < 0.05).

### Analysis of environmental factors influencing the active ingredients and antioxidant activity

#### Principal component analysis (PCA) of environmental factors

**Figure 5:**
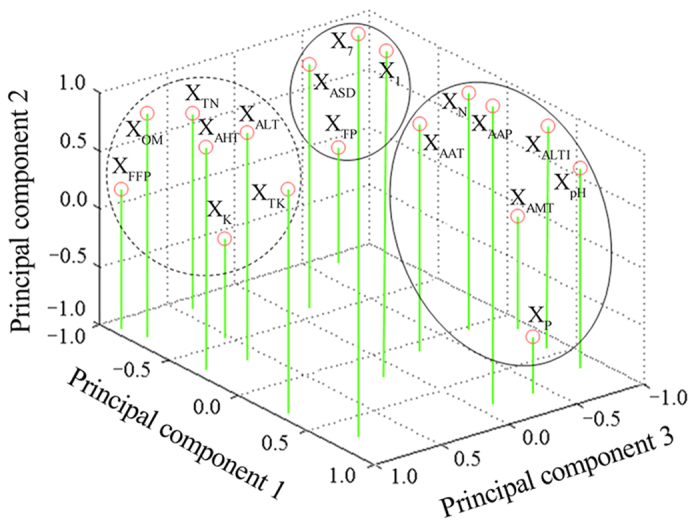
Loading plot generated from principal component analysis (PCA) for each environmental factor. X_N_(mg/kg), rapidly available nitrogen; X_P_(mg/kg), rapidly available phosphorus; X_K_(mg/kg), rapidly available potassium; X_OM_(%), organic matter; X_TN_(%), total nitrogen; X_TP_(%), total phosphorus; X_TK_(%), total potassium; X_pH_, pH; X_AMT_(°C), annual mean temperature; X_1_(°C), january average temperature; X_7_(°C), july average temperature; X_AAT_(°C), annual accumulated temperature(≥10 °C); X_AHT_(°C), annual highest temperature; X_ALT_(°C), annual lowest temperature; X_AAP_(mm), annual average precipitation; X_ASD_(h), annual sunshine duration; X_FFP_(d), frost free period; X_ALTI_(m), altitude.

The PCA was conducted to identify the principal variables from a lot of independent variables (environmental factors). Contribution rate reflects the quantity of original information contained within each factor. The accumulated contribution rate of the first three eigenvalues reached 95.867% (Table 1), which indicates that the first three main components nearly covered total original information of the eighteen environmental factors. Thus, these components can be extracted to obtain the loading level of each environmental factor based on SPSS 19.0 software (Fig. 5). Figure 5 indicated that important environmental factors that effected the first principal component (F_1_) were X_N_ (0.872), X_AAP_ (0.833), X_ALTI_ (0.753), X_pH_ (0.651), X_AAT_ (0.434), X_AMT_ (−0.132), and X_P_ (−0.472). For the second principal component (F_2_), important environmental factors were the X_7_ (0.853), X_1_ (0.815), X_ASD_ (0.757), and X_TP_ (−0.123). The third principal component (F_3_) accounted for a larger proportion in the X_OM_ (0.761), X_TN_ (0.723), X_ALT_ (0.423), X_AHT_ (0.411), X_FFP_ (0.146), X_TK_ (−0.387) and X_K_ (−0.614) than that in other factors. However, its contribution rate was only 1.111%, and the F_3_ was not be considered. Two principal components of environmental factors, F_1_ (X_N_, X_AAP_, X_ALTI_, X_pH_, X_AAT_, X_AMT_, and X_P_) and F_2_ (X_7_, X_1_, X_ASD_, and X_TP_) were thus screened for further analysis.

#### Gray correlation analysis (GCA)

A GCA was conducted between the contents of active ingredients, antioxidant activity, and the principal components of environmental factors (F_1_, F_2_) to deduce primary environmental factors^25^,^26^. The results of GCA (Table 2) demonstrated that environmental factors imposed different degrees of influences on different active ingredients and antioxidant activity: the primary environmental factor for the accumulation of tannin was altitude (X_ALTI_); for the accumulation of total flavonoids, rutin and antioxidant activity, were annual sunshine duration (X_ASD_) and altitude (X_ALTI_); for the accumulation of total phenolics, were annual mean temperature (X_AMT_), annual average precipitation (X_AAP_), and altitude (X_ALTI_).

#### Path analysis (PA)

Although primary environmental factors could be clarified by GCA, the correlations between active ingredient contents, antioxidant activity, and the primary environmental factors remain unclear. So, the correlation between active ingredient contents, antioxidant activity, and the primary environmental factors were further explored by PA, which makes the multiple statistical analyses more rational because the path coefficient possesses directional features^27^. The results of PA (Table 3) in this study demonstrated the effect of primary factors was significant on active ingredient contents and antioxidant activity. X_ALTI_ (altitude) was key environmental factor for the content of tannin, X_ASD_ (annual sunshine duration) and X_ALTI_ (altitude) were key ones for flavonoids, rutin and antioxidant activity, while X_AMT_ (annual mean temperature) and X_ALTI_ (altitude) were key ones for total phenolics. The content of tannin was significantly and negatively correlated to altitude (*P* < 0.05). The flavonoids content, rutin content and antioxidant activity were significantly and positively correlated to annual sunshine duration and altitude (*P* < 0.05). The content of total phenolics was significantly and negatively correlated to annual mean temperature, while the content of total phenolics was significantly and positively correlated to altitude (*P* < 0.05). No significant correlation was observed between the soil factors and the contents of each active substance or antioxidant activity.

The statistical significance demonstrated that *P. fruticosa* samples from Kangding, Sichuan Province (S8) was selected as the most valuable materials due to the higher phytochemical contents and significant antioxidant activity. Furthermore, Kangding, Sichuan Province was proved to be the most suitable region for producing *P. fruticosa* through the comprehensive investigation and analysis of the effect of environmental factors.

### Quality assessment of *P. fruticosa* based on RP-HPLC (reverse phase-high performance liquid chromatography) fingerprint data

#### Similarity analysis (SA)

A standard HPLC fingerprints of 8 batches of samples from different regions of China were obtained under the optimized HPLC conditions (Fig. 1). These herbs showed different chromatographic profiles. After carefully analyzing the fingerprint profiles of these samples, ten common peaks with acceptable heights and good resolution were selected as characteristic peaks for the identification and classification of the *P. fruticosa* samples from different production regions^16^,^19^,^21^,^22^. The relative retention time and relative areas of these constituents (characteristic peaks) were calculated with respect to the reference peak eluting at a retention time of 18.0 min (peak 4). The similarities among the 8 chromatograms were evaluated based on a matrix comprising the relative areas of the 10 constituents of samples 1–8 by the CASE software. The results showed that the similarity of chromatograms of eight samples had a wide range and varied within the range of 0.21–0.89 (Table 4), which indicated that the chromatographic patterns of the samples varied greatly in different production regions.

#### Hierarchical clustering analysis (HCA)

**Figure 6:**
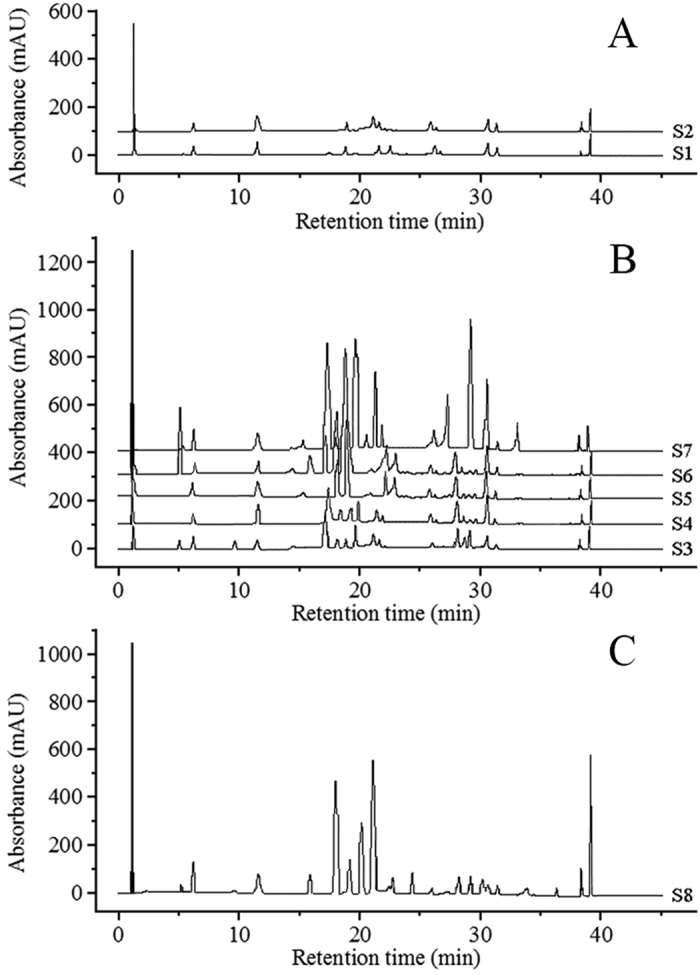
Visual classification for HPLC chromatograms of eight *P. fruticosa* samples from different growing locations.

**Figure 7:**
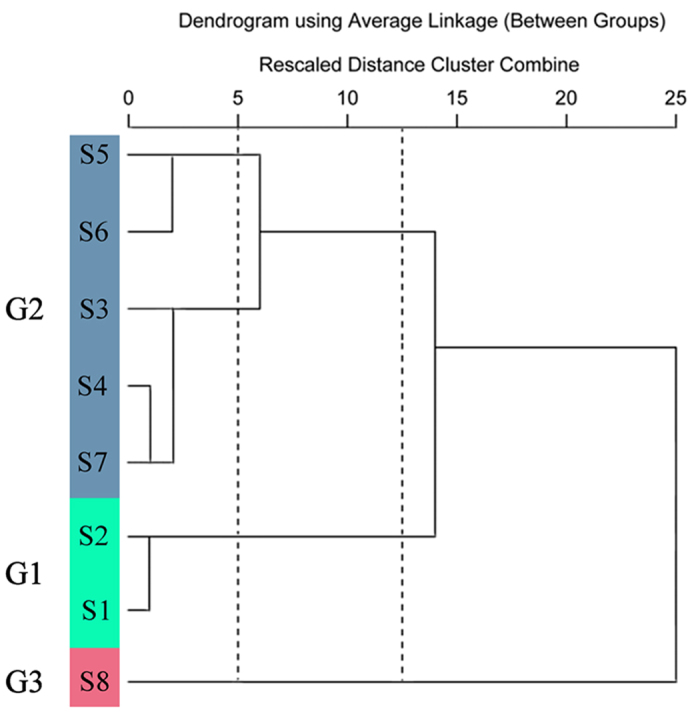
Dendrograms of hierarchical cluster analysis (HCA) for eight *P. fruticosa* samples from different growing locations.

Prior to HCA, we compared the fingerprints visually and simply divided the samples into three distinct groups, namely A, B, and C (Fig. 6). Although it was possible to differentiate chromatograms on the basis of visual comparison, this process was subjective and non-quantitative. HCA can provide a quantitative and objective analysis of fingerprints^20^. HCA was performed according to the relative peak areas of ten constituents and a dendrogram was acquired (Fig. 7). At the rescaled distance of 12.5, HCA can divide the eight samples from different locations into three groups consistent with the results of visual comparisons. S1 and S2 were merged into a group G1; S3, S4, S5, S6, and S7 were grouped in G2; and S8 was found in G3.

The correlation coefficients of each chromatogram within groups G1, G2 and G3 corresponding to software-generated group simulated mean chromatograms, and the correlation coefficients between these simulated mean chromatograms were shown in Table 5. The chromatograms within a particular group were generally consistent. Correlation coefficients for each chromatogram which were classified into a particular group to the corresponding simulative mean were higher than 0.90. However, the chromatograms within a particular group were markedly different from the chromatograms in different groups, and the differences in the similarity values for the three groups (Table 5) reflected the HCA-generated data. Meanwhile, the correlation coefficient between the software-generated group simulated mean chromatograms was lower than 0.65, and ANOVA investigations indicated the difference between groups was significant (*P* = 0.041). These results demonstrate that HCA can distinguish *P. fruticosa* species with the same origin from different districts.

#### Discrimination analysis (DA)

Ten common peaks were selected from the fingerprints, thereby creating 10 variables. However, not all these variables are of value to establish discriminant function. The DA will generate discriminant functions only by use of valuable variables. Two types of discriminant functions [equations (1) and (2)] were obtained using the SPSS software (SPSS for Windows 19.0, SPSS Inc., USA).

Canonical discriminant function:


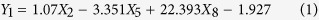



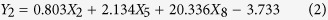


Discrimination standard:


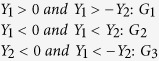


Fisher’s discrimination function:


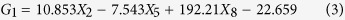



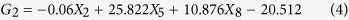



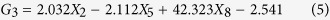


Discriminant standard: each sample has three functional values and is assigned to the group corresponding to the highest of these function values. G1 [equation (3)], G2 [equation (4)] and G3 [equation (5)] denote the samples from groups G1, G2 and G3 respectively, and X denotes the variable.

**Figure 8:**
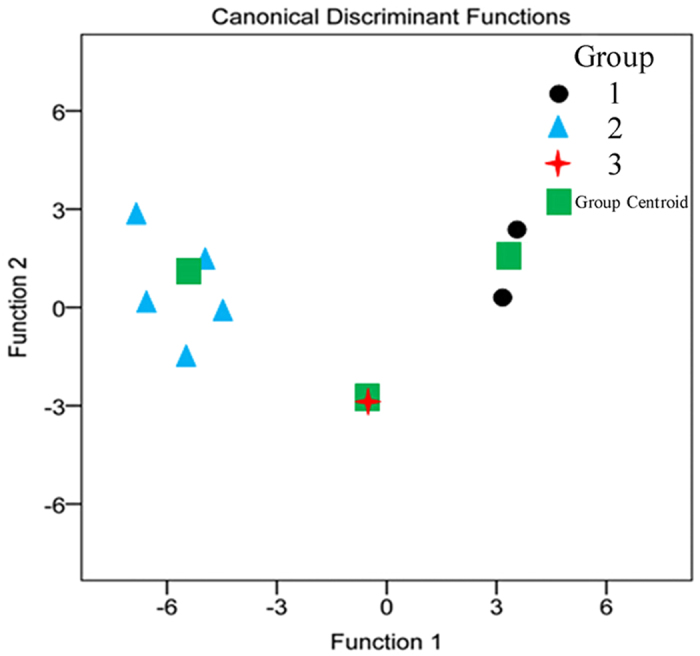
Discrimination analysis (DA) for eight *P. fruticosa* samples from different growing locations.

Only three variables, corresponding to the areas of peaks 2, 5 and 8, were used to generate the discriminant functions. In order to place an unknown sample, the values of the three variables are inserted into the equations, and the unknown sample was grouped according to the discriminant standard value obtained. Use of the three most discriminating variables enable test samples belonging to groups G1, G2 and G3 to be classified with 100% accuracy. The well-resolved DA plots for the three groups are shown in Fig. 8. DA approach supported a division of the 8 samples into 3 major groups for which HPLC SA confirmed the considerable variation in the active substances and quality of *P. fruticosa* from different growing locations. Furthermore, the samples from Kangding, Sichuan Province (S8) was separately assigned into a special group Group 3 (Fig. 8), and also far away from Group 1 and 2. There was a huge overlap between the center of the samples contained by Group3 (S8) and group centroid, indicating that S8 was in high quality.

## Discussion

### Variation in active substance contents and antioxidant activity

Altitude, temperature, illumination, and moisture are important factors to influence the metabolism and accumulation of secondary metabolites^28^. Environmental differences (such as altitude, temperature, illumination, precipitation, humidity, soils) in different production locations contribute to the differences in active ingredient contents and antioxidant activity of medicinal plants^9^,^16^,^19^. In this study, significant differences were observed in the contents of the chemical compositions and antioxidant activity of *P. fruticosa* leaves obtained from different growing locations (Figs 1~4). The contents of tannin in the leaves of *P. fruticosa* planted in S8 (Kangding, Sichuan) were higher than those planted in other locations, followed by S1 (Mei county, Shaanxi). The contents of total flavonoids were high in those planted in S8 (Kangding, Sichuan), followed by S7 (Ningchi, Tibet). Li *et al*. also discovered that the contents of total flavonoids in *P. fruticosa* leaves from different environments displayed great differences^29^, which agrees with our findings. The contents of rutin were also high in S8 (Kangding, Sichuan), followed by S7 (Ningchi, Tibet). The contents of total phenolics were high in S6 (Shangri-la, Yunnan), followed by S8 (Kangding, Sichuan). The antioxidant activity was high in S8 (Kangding, Sichuan), followed by S2 (Diebu, Gansu). The results indicate that the contents and properties of active substances are closely related to the growing locations, and are determined by environmental factors of the growing locations. Integrating the active substance contents, antioxidant activity ability and the effect of environmental factors, *P. fruticosa* from Kangding, Sichuan Province (S8) was selected as the most valuable material, and Kangding, Sichuan Province was considered as the most suitable region for producing *P. fruticosa*. The rich variation in chemical compositions and antioxidant activity may lead to significant differences in effectiveness as medicines, functional foods and nutritional supplements. HPLC fingerprint SA coupled with HCA and DA were further introduced to establish an effective method for the accurate classification and quality assessment of *P. fruticosa*. This method performed well for the quality evaluation of *P. fruticosa* samples and also confirmed that S8 had high quality.

### Altitude and active substance contents

Altitude is an overall reflection of multiple ecological factors, such as temperature, humidity, and solar radiation. Many studies have confirmed that the contents of flavonoids are positively correlated to the altitude of the growing location. For example, in *Leontodon* species, analyses of samples originating from different altitudes (13 samples of *Leontodon helveticus* Mérat from 1600–2950 m, 19 samples of *Leontodon autumnalis* L. from 30–2075 m, and 61 samples of *Leontodon hispidus* L. from 40–2550 m) showed a significant positive correlation between altitudes of collection sites and total contents of flavonoids^28^. Dong *et al*. selected *Eucommia ulmoides* that is a medicinal plant from six altitudes (550 m, 690 m, 780 m, 845 m, 950 m and 1180 m) as study materials and found that altitude was significantly and positively correlated to the contents of flavonoids (*P* < 0.05)^9^. Ghasem e*t al*. studied the influence of environmental factors on flavonoids contents of walnut (*Juglans regia* L.) green husks and found the good correlation coefficient was existed between the flavonoid contents and collection altitudes^30^. The altitude and other information about locales of sample collection were shown in Table 6. Similarly, in this study, the altitude in Kangding is higher than other locations, the contents of total flavonoids were the highest, and significantly different from other locations (Fig. 2). GCA and PA showed that maximum degree of correlation was found between altitude and the contents of total flavonoids, and positive relationship existed (Tables 2 and 3). Flavonoids contain ortho-dihydroxylated structure, which exhibits the characteristics of absorbing ultraviolet, and scavenging free radicals. Significant positive correlation between the contents of total flavonoids and altitude are responses of the *P. fruticosa* species distributed at higher altitude to the strong ultraviolet radiation. Also, UV-absorbing compounds such as flavonoids and phenolic acids have been proven to be important for plants to protect their vegetative organs from harmful UV radiation^13^. By contrast, the total amount of flavonoids of some medicinal plants, such as *Buxus sempervirens* L., decreased with increased altitude^31^. The reduction of flavonoids in *B. sempervirens* leaves with altitude might be a consequence of an increased synthesis of wax in response to the decrease in temperature^32^ since both, cuticular waxes and flavonoids, are derived from acetyl-CoA^33^.

### Temperature and active substance contents

The production of secondary metabolites by temperature stress is the expression of self defense mechanism of medicinal plants. Recently a significant increase of the biosynthesis of phenolics was demonstrated for plants growing in a low temperature regime^34^. Similarly, in this study, annual mean temperature was significantly and negavely correlated to the contents of total phenolics. This result agreed with the previous researches for total phenolics contents involved in other medicinal plants. For example, in walnut (*Juglans regia* L.), a negative correlation was found between temperature and total phenolic contents^30^. Albert *et al*. reported that enhanced UV-B radiation is probably not the key factor triggering shifts in the phenolic composition in *Arnica montana* L. cv. ARBO grown at higher altitudes but rather temperature, which displays negative correlation with the phenolics^35^. Reyes *et al*. studied total phenolics during development of *Solanum tuberosum* L. in cultivars grown in Texas and Colorado and found cooler temperatures in Colorado favored about a 1.4-times higher total phenolics content than in Texas-grown tubers^36^. The present study also showed phenolic compounds increased with altitude, similar the trends as the results for *Buxus sempervirens* obtained in a previous study^31^. Usually a decrease of the mean temperature of 0.55 °C per 100 m of altitude is observed^37^. Hence, the contents of phenolic compounds increased with the decrease of temperature. However, temperature is positively correlated to the contents of active ingredients in some plants. The contents of hypericin in *Hypericum perforatum* L. increased with temperature^38^. The content of anthocyanin in sugarcane that was under high temperature obviously increased^39^. Plants in alpine habitats are exposed to many environmental stresses, in particular temperature and radiation extremes. Another laboratory studies confirm that the biosynthesis of certain phenolic compounds is increased under increased UV radiation, whereas shading leads to a decrease in the biosynthesis of these compounds^40^. Therefore, plant could have a chemical adaption to the alpine environment, indicating that the impacts of ecological factors on the secondary metabolites are related to their chemical types, structures and characteristics.

### Sunshine duration and active substance contents

Illumination also affects the synthesis and accumulation of secondary metabolites in medicinal plants. The increase of illumination time can increase the contents of secondary metabolites. For example, the amount of flavonoids in *Arabidopsis* increased after long time illumination^41^. The contents of ginsenogsides in *Panax quinquefolius* L. were positively correlated to the annual sunshine duration^42^. Dong *et al*. investigated the influence of environmental factors on the contents of secondary metabolites in the leaves of *Eucommia ulmoides* and found that annual sunshine duration was significantly and positively correlated to the contents of geniposidic acid (*P* < 0.05)^9^. In this study, PA showed that the contents of active substances (tannin, total flavonoids, rutin, and total phenolics) were highly associated to annual sunshine duration, positive correlation between them was observed (Table 3). The results were also verified in the study on production of active substances of other medicinal plant such as *Sinopodophyllum hexandrum*^16^. These data indicated that annual sunshine duration is a key environmental factor to the synthesis and accumulation of active substances. *P. fruticosa* is a heliophilous plant, locations with long time sunshine would be favorable for its secondary metabolism, resulting in adequate substrate to catalyze the synthesis of the active substances and thereby increasing their contents.

### Precipitation and active substance contents

In this study, the contents of tannin, rutin and total phenolics were negatively correlated to the annual average precipitation. This finding was consistent with the previous research results about other active substances. For example, the quinine content of *Cinchona ledgeriana* (Howard) Bern. Moens ex Trimen was higher under drought conditions and significantly lower under excessive soil humidity, even to the point of not being synthesized at all^16^. The atropine content of *Anisodus carniolicoides* (C.Y. Wu & C. Chen) D’Arcy & Zhi Y. Zhang could be as high as 1% under dry conditions, which is 2.5-times greater than that obtained under humid conditions (0.4%)^16^. In the meantime, the study showed that the contents of total flavonoids were positively correlated to annual average precipitation. Although the contents of tannin, rutin and total phenolics were negatively correlated to the annual average precipitation, and the contents of total flavonoids were positively correlated to annual average precipitation, the impacts of annual average precipitation on each secondary metabolite were not significant (Table 3). Therefore, precipitation is not a limited factor to the synthesis and accumulation of secondary metabolites for *P. fruticosa*. Additionally, 500 mm annual precipitation can meet the demand of the growth of *P. fruticosa* according to the biological features and the demands for environmental conditions. In the field investigation, the annual average precipitations in 8 locations are all over 500 mm, they all can meet the moisture demand from the growth of *P. fruticosa*. This could explain why the influence of annual average precipitation was not significant on active substances of *P. fruticosa*.

### Soil and active substance contents

Some researchers have stated that the variations in the active ingredient contents of the plants are related to soil fertility. For example, the leaf polyphenol content of young greenhouse tomato plants was increased considerably in response to low N availability^43^. The saponin contents of *Panax quinquefolius* were increased by 27.86% by the repeated applications of high-quality organic fertilizer and the functionality of potassic fertilizer was the same as that of a total nutrient admixture in terms of saponin contents^44^. Liu *et al*. reported that organic matter was significantly and positively correlated with the active substances of *Sinopodophyllum hexandrum*^16^. In this study, no correlation was observed between the soil factors and the contents of each secondary metabolite of *P. fruticosa*. GCA that screened primary factors for active substances demonstrated that no soil factors were included in primary factors, indicating soil factors were not main drive force contributing the contents of active substances. Similarly, Liu *et al*. also found the soil factors contributed less to the active ingredient contents in *Sinopodophyllum hexandrum*^16^ than did the climate factors. The field investigation found all the growing locations and soil were well protected and managed under national conservation policy. Soil with abundant nutrients could meet the growth and development of *P. fruticosa*. Compared with soil factors, variation of climatic factors played an important role in the accumulation of active substances, which may explain the reason why no correlation existed between the soil factors and the contents of active substances of *P. fruticosa*. A deep reason should be explored by the investigation of metabolism of soil microorganism, soil trace elements and key enzymes combined with molecular techniques.

### Environmental factors and antioxidant activity

Many reports strongly suggested that environmental conditions influenced the antioxidant properties of the plants. For instance, Yu *et al*.^45^ reported that significant effects of growing conditions, including number of hours exceeding 32 °C, on the antioxidant properties of Akron, a hard red winter wheat variety. The average temperature at growing locations was shown to have effects on the antioxidant properties of strawberries^46^. Mpofu *et al*. reported that location effects were highly significant (*P* < 0.0001), and wheat grown in Winnipeg with the highest altitude had the highest antioxidant activity^47^. Similarly, altitude showed significant and positive correlation to the antioxidant activity in this study. These data suggest potential influences of growing conditions on the antioxidant properties of *P. fruticosa* and the possibility of producing a selected wild species with a desired antioxidant property by optimizing growing conditions. The significant effects of environment must, therefore, be considered. However, few specific data are available in the literature on the relationship between environmental factors and antioxidant activity of *P. fruticosa*. More research is required to further investigate the cause of environmental factor effects using a greater sample of both field locations and varieties. Going forward, we would conduct further studies in cells and living bodies to fully reflect the antioxidant properties of *P. fruticosa*, which could not be achieved by the DPPH scavenging capacity assay and FRAP assay carried out above.

### Quality assessment of *P. fruticosa*

Traditional Chinese medicine (TCM), which has a 5000-years history of application, is still in wide demand^48^. TCMs are being closely examined for the development of novel pharmaceuticals^49^. However, quality control of TCM has always been a bottleneck for their adoption worldwide because of their complexity, the presence of unknown components and lack of quality control. Chromatographic fingerprint analysis has been introduced as a rational strategy for the assessment of complex TCMs. Unlike the traditional practice that one or more compounds were selected as active markers for identification and quality assessment, fingerprint relies on the inherent relationship of multiple compounds and displays the chemical pattern of TCM. Among available quality control methods, chromatographic fingerprint has gained more and more attention recently. In this study, HPLC fingerprint coped with chemometric methods were applied to deal with the classification for the quality assessment of *P. fruticosa* from different regions of China. The results showed that the eight samples were divided into 3 clusters. Two types of discriminant functions were generated using three selected predictor variables and the ratio of discrimination was 100%. The results of HPLC fingerprint SA, HCA, and DA in this study were in agreement, and the techniques can identify and classify the same species from different regions and performed well for the quality assessment of *P. fruticosa*. Also, it can be used to compare and control other natural products prepared from them. Many similar studies on other medical plants have been published for the quality evaluation^20^,^21^,^22^,^23^. The quality of herbal drugs is affected by many composite factors. Climatoecological type, symbiosis ecotype, and soil ecotype in growing locations are biologically essential to produce high-quality geoauthentic herbal drugs^9^. For the widespread plant species, because the large span in geological zones, differences in chemical constituents within the same plant species would occur. *P. fruticosa* belongs to one of widespread plant species, distributed in the Asia, Europe and North America. Currently, the influence of environmental factors in other locations on active substances of *P. fruticosa*, and its quality assessment are less studied. Further research include more samples from other regions of China and other countries should be carried out. The present study provides meaningful information for the collection and application of wild *P. fruticosa* herbs in both healthcare and the food industry. Moreover, although this present study relates only to *P. fruticosa*, the methodology has wider relevance for quality assessment of other edible and medicinal plants.

## Methods

### Plant materials and soil samples

**Figure 9:**
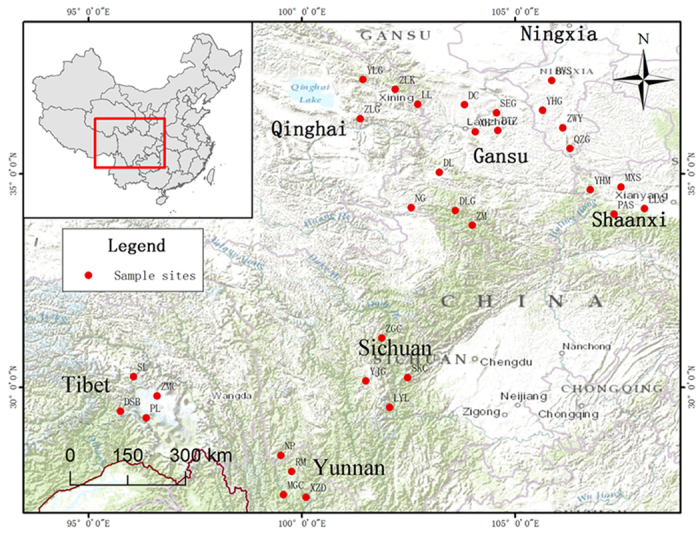
Growing locations of *P. fruticosa* samples involved in seven provinces of China sampled for this study. Maps generated using ArcGIS 10.0 (ESRI Inc. 2014; http://www.esri.com/).

*P. fruticosa* were collected from eight representative locations located in seven provinces of China during 10–25th July in 2014 (Fig. 9). Specifically, a total of eighty healthy specimens from four populations (each population was separated geographically by at least 30 km, and 5 m for adjacent individuals) with similar growth stature were collected in every test region, as shown in Table 7. Matured leaves were picked up respectively from four directions (north, south, east, and west) of three positions (up, middle and low part) of the plant and then mixed as one sample for each test location, thereby obtaining eight test samples. All samples were dried under the vacuum at 40 °C. After grinding into powders, the samples were stored in the dark at −20 °C before further use. Simultaneously, soil rhizosphere were collected and treated for each study site, thereby obtained eight soil samples from eight study sites for the measurement of soil factors. Voucher specimens from all populations were identified by professor Jianjun Liu of Northwest A&F University and were deposited at the Herbarium of Northwest A&F University (WUK0781792-0781823).

### Related data of environmental factors

Soil samples were used to determine the key soil parameters including rapidly available nitrogen (X_N_), rapidly available phosphorus (X_P_), rapidly available potassium (X_K_), organic matter (X_OM_), total nitrogen (X_TN_), total phosphorus (X_TP_), total potassium (X_TK_), and pH (X_pH_) using the modified soil chemistry analysis methods described by Sparks *et al*.^50^. Related data of climate factors including annual mean temperature (X_AMT_), january average temperature (X_1_), july average temperature (X_7_), annual accumulated temperature (≥10 °C) (X_AAT_), annual highest temperature (X_AHT_), annual lowest temperature (X_ALT_), annual average precipitation (X_AAP_), annual sunshine duration (X_ASD_), and frost free period (X_FFP_) in the 50 years (1964~2013) was collected from local meteorological bureaus (stations) for the eight study sites. The environmental factors of the eight study sites were summarized in Table 6.

### Preparation of the extracts

Considering the impact of various factors, the extraction process was optimized using a response surface method^51^. The optimized extraction process was as follows: ethanol concentration, 70%; extraction temperature, 45 °C; extraction time, 2 h; extraction times, three; and liquid-solid ratio, 15:1. Each powdered sample (5.0 g) was treated as described in the optimized extraction process. The obtained filtrates were evaporated at 40 °C under vacuum using a rotary evaporator, and were then stored in the dark at 4 °C for further use. Extracts were diluted if necessary. Each sample was performed in triplicate.

### Measurement of tannin

Tannin contents were determined using the Folin-Denis method^52^. 1.0 mL of the diluted samples (2 mg/mL) was transferred to a 25 mL calibration flask to which were added 1.0 mL of Folin-Denis reagent and 5 mL of sodium carbonate (1 mol/L). The solution was diluted to 25 mL by the addition of methanol. After incubating for 30 min at room temperature, the absorbance at 720 nm was measured against a blank. Tannin acid (1 mg/L to 10 mg/L) was used for the standard curve calibration. All measurements were performed in triplicate.

### Measurement of total flavonoids

Total flavonoids contents were determined by sodium nitrite-aluminum nitrate colorimetric method^53^. Sample solution (1.0 mL, 2 mg/mL) was transferred into 25 mL volumetric flasks, then 0.3 mL NaNO_2_ (5%) was added and held for 6 min. Next, 0.3 mL Al(NO_3_)_3_ (10%) was added and held for another 6 min. Finally, 4 mL NaOH (1 mol/L) was added and the solution was diluted to 25 mL with 70% ethanol solution. After 30 min of incubation at room temperature, the absorbance at 510 nm was measured against a blank. Rutin (4 mg/L to 40 mg/L) was used for the standard curve calibration. All measurements were performed in triplicate.

### Measurement of total phenolics

The total phenolic contents were determined using a modified Folin-Ciocalteau colourimetric method^54^. Sample solution (1 mL, 2 mg/mL) was transferred to a 25 mL calibration flask, 0.5 mL of Folin-Ciocateu reagent and 2.5 mL of sodium carbonate (1 mol/L) was added and final volume was marked with methanol. After 30 min of incubation at 30 °C, the solution was centrifuged at 4,000 rpm (10 min) and the absorbance at 760 nm was measured against a reagent blank. Gallic acid (0 mg/L to 6 mg/L) was used for the standard curve calibration. Three replicates were made for each sample.

### RP-HPLC analysis

Sample solution (1 mg/mL) was filtered through a 0.22 μm microporous filtering film and were then separated by RP-HPLC at ambient temperature to obtain chromatograms^55^. The amounts of rutin were also quantified by RP-HPLC. The contents of rutin were identified and calculated by a comparison of the relative retention time (RRT) and relative peak areas (RPAs) with those of the standards. HPLC analyses were conducted on an Agilent Series 1260 liquid chromatograph, equipped with a quaternary gradient pump, a variable wavelength detector system and a reversed-phase SB-C18 column (5 μm, 4.6 × 250 mm, Agilent, USA). The mobile phase consisted of water with 0.5% acetic acid (mobile phase A) and methanol with 0.5% acetic acid (mobile phase B). The flow rate was 1 mL/min. The gradient program was as follows: 0–15 min, 25% to 35% B; 15–30 min, 35% to 60% B; 30–40 min, 60% to 100% B; 40–45 min, hold B at 100%. The injection volume and detect wavelength were 20 μL and 360 nm, respectively^56^,^57^. Analyses were performed in triplicate.

### DPPH radical scavenging activity

DPPH assay has been widely used for the determination of antioxidant activity of pure antioxidant compounds as well as of different plant extracts, a lower IC_50_ representing stronger antioxidant capacity^24^. IC_50_ obtained by interpolation from linear regression analysis is the effective sample concentration at which DPPH radicals is scavenged by 50%. A slightly modified DPPH method was used to determine the radical scavenging property of *P. fruticosa* leaves^58^. Amounts of 2 mL of the tested samples (1–100 μg/mL) and the positive controls (rutin, 1–70 μg/mL) were mixed with 2.0 mL of 0.1 mol/L DPPH in methanol. The mixture was vortexed thoroughly and allowed to stand in the dark for 30 min. The absorbance was measured at 517 nm spectrophotometrically against a blank. All measurements were performed in triplicate. DPPH free radical scavenging activity (SA) can be expressed with the following equation (6):


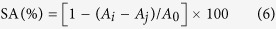


where A_i_: absorbance of tested samples (2 mL) mixed with DPPH (2 mL); A_j_: absorbance of tested samples (2 mL) mixed with methanol (2 mL); A_0_: absorbance of methanol (2 mL) mixed with DPPH (2 mL).

### Ferric reducing antioxidant power (FRAP) assay

In this assay, the colour changed from yellow to green depending on the reducing power of the sample. Fe^3+^/ferricyanide complex was reduced to the ferrous form by reductants in the solution. Thus, Fe^2+^ can be monitored as the reducing power index by measuring the absorbance. The reducing capacity of the extract was determined by the method of Oyaizu^59^. Varying concentrations of the extracts in the methanol (2.5 mL, 50–500 μg/mL) were mixed with phosphate buffer (2.5 mL, 0.2 mol/L, pH 6.6) and potassium ferricyanide (2.5 mL, 1%), and incubated at 50 °C for 20 min. Aliquots (2.5 mL) of 10% trichloroacetic acid were added to the reaction mixture, which was then centrifuged at 4,000 rmp (10 min). The upper layer of the solution (2.5 mL) was mixed with distilled water (2.5 mL) and ferric chloride (0.5 mL, 0.1%). The absorbance was measured at 593 nm. The FRAP results were expressed in terms of micromoles trolox equivalent per gram dry weight of samples (μmol equiv. Trolox/g). All measurements were performed in triplicate.

### Data analyses

**Figure 10:**
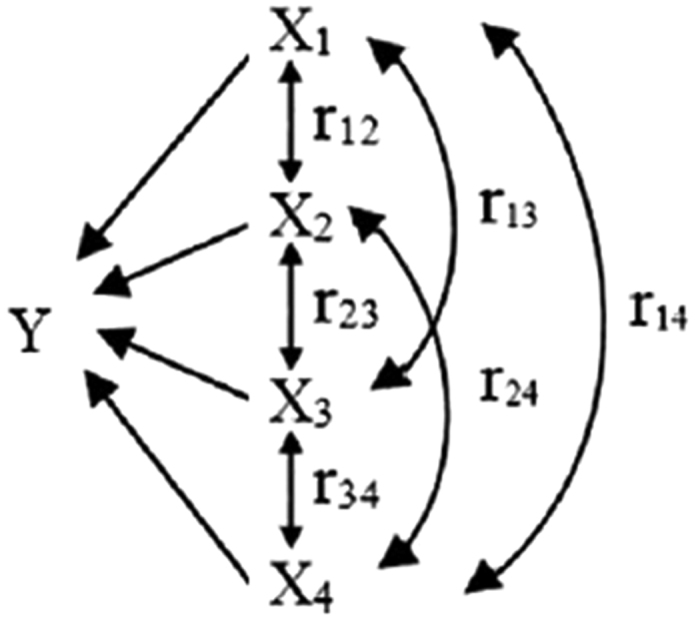
Network of path analysis. X_1_, X_2_, X_3_ and X_4_ are independent variables; Y is dependent variable. r_ab_ represents the correlation coefficient between independent variables a and b.

Three methodologies were performed step-by-step to analysis systematically the influence of environmental factors on the active substance contents and antioxidant activity of *P. fruticosa* leaves, which also represented a quantitative comparative analysis of the dynamic process of plant and the environment. Firstly, principal component analysis (PCA) was carried out using SPSS software (SPSS for Windows 19.0, SPSS Inc., USA). PCA has the strong advantage of significantly reducing the dimension of the complex data while preserving most of the variance within by using dependencies among large numbers of variables without requiring knowledge of the data set in order to visualize high dimensional data and identify the most important variables^60^. PCA in the present study was used to find out principal components of environmental factors. Secondly, gray correlation analysis (GCA) between the contents of active substances, antioxidant activity and the principal components of environmental factors obtained by PCA were conducted by DPS7.5 software (Date Processing System, Science Press, China) to screen primary factors from principal components of environmental factors. The result of GCA reflects the close degree of the relationship between principal behavior factors and other factors, to deduce primary factors and secondary factors based on the gray correlation degrees of different factors^9^. High correlation degree indicates high influence of the factor to the accumulation of active substances and antioxidant activity^25^,^26^. GCA is usually applied in the indefinite system, including small sampling and poor data information system, in which limited information is available, others are not known^25^,^26^. Therefore, it is suitable to be used for the analysis of the data obtained in this study. Thirdly, path analysis (PA) deals with the quantitative relationship between dependent and independent variables to explain the relative significance of each factor to the dependent variables^9^,^27^. Figure 10 showed the path network of four independent variables included in PA. PA can determine whether the effect of X_i_ on Y is significant or not, and can identify not only the direct effect of X_i_ on Y (b_i_, X_i_—Y) but also indirect effect of X_i_ on Y through X_j_ (r_ij_b_j_, X_i_—X_j_—Y, i≠j). Thereby, the correlation coefficient (r_iy_) contains the direct path coefficient (b_i_, X_i_—Y) and the indirect path coefficient (r_ij_b_j_, X_i_—X_j_—Y, i≠j) 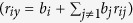. PA was conducted by statistical software SAS 9.1 (SAS Institute, Cary, NC, USA) to evaluate correlation between the active ingredients, antioxidant activity and primary environmental factors obtained by GCA. Environmental factors were used as independent variables, and active ingredients and antioxidant activity were used as dependent variables in each test. Furthermore, in order to further evaluate the quality of *P. fruticosa* from different growing locations, another three methods viz. HPLC fingerprint analysis coupled with chemometric methods were applied. Firstly, HPLC fingerprint similarity analysis (SA) was performed using Computer Aided Similarity Evaluation software (CASE 2004, Zhejiang University, Hangzhou, China) as recommended by the Chinese Pharmacopoeia Committee. The software is used for evaluating similarities between different chromatograms based on the cosine values of vectorial angel^61^. The cosine values of the two chromatograms approaching 1 means they are highly similar. This software was also used to compute the mean chromatogram as a representative standard chromatogram for a group of chromatograms. The standard HPLC fingerprint is set up with the median of all chromatograms^62^,^63^,^64^. Subsequently, hierarchical clustering analysis (HCA) and discrimination analysis (DA) were performed using SPSS 19.0^19^,^20^. The ‘average linkage between groups’ method was applied and the Pearson correlation was selected as a measurement^65^. DA can be used to build a predictive model of the group membership based on observed characteristics in each case. This procedure can generate a discrimination function or a set of discriminant functions from the samples with known membership based on linear combinations of the predictor variables that provide the best discrimination among the groups. The functions can be applied to discriminate and classify new cases with measurements for the predictor variables but with unknown group membership^20^. A detailed description/theory of statistical methods/models was provided in supplementary Text S1. Normalization of data was performed by the Z score transformation normalization method if necessary^66^.

The results were presented as the mean value ± SD (standard deviation). The data was analyzed by one-way ANOVA (Analysis of Variance) followed by Duncan multiple comparison (*p* < 0.05) based on SPSS software (SPSS for Windows 19.0, SPSS Inc., USA).

## Additional Information

**How to cite this article**: Liu, W. *et al*. Influence of Environmental Factors on the Active Substance Production and Antioxidant Activity in *Potentilla fruticosa* L. and Its Quality Assessment. *Sci. Rep*. **6**, 28591; doi: 10.1038/srep28591 (2016).

## Supplementary Material

Supplementary Information

## Figures and Tables

**Table 1:** Eigenvalues and cumulative contribution rates of principal components.

Ecological factors	Principal components	Eigenvalues	Contribution rates (%)	Cumulative contribution rates (%)
X_N_	F_1_	8.95	54.782	54.782
X_P_	F_2_	7.784	39.974	94.756
X_K_	F_3_	3.339	1.111	95.867
X_OM_	F_4_	1.734	0.915	96.782
X_TN_	F_5_	0.423	0.807	97.589
X_TP_	F_6_	1.725	0.613	98.202
X_TK_	F_7_	0.68	0.572	98.774
X_pH_	F_8_	0.437	0.4105	99.184
X_AMT_	F_9_	0.342	0.377	99.561
X_1_	F_10_	0. 175	0.3135	99.875
X_7_	F_11_	0.072	0.1065	99.981
X_AAT_	F_12_	0.057	0.0065	99.988
X_AHT_	F_13_	0.041	0.004	99.992
X_ALT_	F_14_	0.033	0.0024	99.994
X_AAP_	F_15_	0.024	0.0021	99.996
X_ASD_	F_16_	0.022	0.0015	99.998
X_FFP_	F_17_	0.018	0.001	99.999
X_ALTI_	F_18_	0.011	0.001	100

X_N_(mg/kg), rapidly available nitrogen; X_P_(mg/kg), rapidly available phosphorus; X_K_(mg/kg), rapidly available potassium; X_OM_(%), organic matter; X_TN_(%), total nitrogen; X_TP_(%), total phosphorus; X_TK_(%), total potassium; X_pH_, pH; X_AMT_(°C), annual mean temperature; X_1_(°C), january average temperature; X_7_(°C), july average temperature; X_AAT_(°C), annual accumulated temperature(≥10 °C); X_AHT_(°C), annual highest temperature; X_ALT_(°C), annual lowest temperature; X_AAP_(mm), annual average precipitation; X_ASD_(h), annual sunshine duration; X_FFP_(d), frost free period; X_ALTI_(m), altitude.

**Table 2:** Gray correlation coefficients between the active ingredients, antioxidant activity and related environmental factors.

Environmental factors	Correlation degree				
	Tannin	Total flavonoids	Rutin	Total phenolics	IC_50_
X_N_	0.313	0.456	0.501	0.444	0.478
X_AAP_	0.351	0.511	0.421	0.602	0.507
X_ALTI_	0.717	0.758	0.661	0.724	0.776
X_pH_	0.471	0.376	0.357	0.376	0.444
X_AAT_	0.402	0.413	0.364	0.461	0.305
X_AMT_	0.357	0.301	0.462	0.598	0.287
X_P_	0.316	0.406	0.456	0.336	0.321
X_7_	0.579	0.432	0.477	0.458	0.429
X_1_	0.481	0.338	0.328	0.294	0.328
X_ASD_	0.357	0.674	0.733	0.328	0.613
X_TP_	0.322	0.329	0.353	0.422	0.515

X_N_, X_P_, ···, X_ALTI_ was performed in the Table 1. IC_50_ was used to indicate antioxidant activity.

**Table 3:** Path analysis between the active ingredient contents, antioxidant activity and primary factors.

Items	Primary factors	Direct Path coefficient	Indirect Path coefficient					Significance level(P-value)
			Total	→X_AMT_	→X_AAP_	→X_ASD_	→X_ALTI_	
Tannin	X_AMT_	0.447	−0.362		−0.398	0.673	−0.637	0.229
	X_AAP_	−0.821	0.533	0.081		−0.413	0.324	0.327
	X_ASD_	0.541	0.202	0.230	−0.169		0.141	0.073
	X_ALTI_	−0.739	0.210	−0.045	0.001	0.254		0.006
Total flavonoids	X_AMT_	0.422	0.473		0.062	0.142	0.269	0.272
	X_AAP_	0.501	0.322	0.360		−0.368	0.322	0.345
	X_ASD_	0.916	−0.837	−0.442	0.126		−0.521	0.009
	X_ALTI_	0.569	0.334	0.976	0.188	−0.830		0.000
Rutin	X_AMT_	0.851	−0.143		0.508	−0.775	0.124	0.073
	X_AAP_	−0.368	−0.315	−0.683		0.135	0.233	0.062
	X_ASD_	0.711	−0.436	0.227	−0.543		−0.12	0.012
	X_ALTI_	0.413	0.529	0.124	0.264	0.141		0.000
Total phenolics	X_AMT_	−0.429	−0.334		0.380	−0.53	0.576	0.006
	X_AAP_	−0.501	0.322	0.268		−0.168	0.222	0.109
	X_ASD_	0.633	0.281	0.324	0.388		−0.431	0.302
	X_ALTI_	0.736	−0.425	−0.315	−0.376	0.266		0.015
IC_50_	X_AMT_	−0.513	0.796		0.612	0.430	−0.246	0.461
	X_AAP_	0.808	−0.316	0.108		−0.185	−0.239	0.248
	X_ASD_	−0.602	−0.319	0.065	0.272		−0.656	0.003
	X_ALTI_	−0.635	0.241	0.768	−0.651	0.124		0.000

Primary factors including X_AMT_, X_AAP_, X_ASD_, X_ALTI_ were performed in the Table 1. IC_50_ was used to indicate antioxidant activity, a lower IC_50_ representing stronger antioxidant capacity.

**Table 4:** Similarity of the chromatograms of *P. fruticosa* samples from eight locations.

No.	S1	S2	S3	S4	S5	S6	S7	S8
S1	1.00							
S2	0.91	1.00						
S3	0.67	0.61	1.00					
S4	0.73	0.57	0.79	1.00				
S5	0.81	0.70	0.68	0.85	1.00			
S6	0.76	0.32	0.39	0.66	0.53	1.00		
S7	0.34	0.50	0.53	0.41	0.68	0.62	1.00	
S8	0.27	0.46	0.38	0.52	0.21	0.31	0.56	1.00

S1, Mei county, Shaanxi; S2, Diebu, Gansu; S3, Huzhu, Qinghai; S4, Jingyuan, Ningxia; S5, Yongdeng, Gansu; S6, Shangri-la, Yunnan; S7, Ningchi, Tibet; S8, Kangding, Sichuan.

**Table 5:** Correlation coefficients between individual chromatograms within a group and the group simulative mean chromatogram, and between the group simulative mean chromatograms.

Group	G1	G2	G3
G1	0.936 ± 0.023^a^	0.631^b^	0.512^b^
G2		0.944 ± 0.005^a^	0.645^b^
G3			0.999 ± 0.000^a^

^a^Correlation coefficient of individual chromatograms to the simulative mean chromatogram of the corresponding group.

^b^Correlation coefficient between simulative mean chromatograms.

**Table 6:** Main environmental factors of eight locations throughout China.

Items	S1	S2	S3	S4	S5	S6	S7	S8
X_N_	6.31 ± 0.03 abcd	28.12 ± 0.02 cde	32.65 ± 0.04 abc	8.09 ± 0.07 ab	7.43 ± 0.04 abc	8.13 ± 0.03 abcd	32.21 ± 0.02 cde	35.82 ± 0.03 abc
X_P_	6.42 ± 0.06 ab	7.58 ± 0.05 a	8.56 ± 0.02 b	11.37 ± 0.15 de	8.25 ± 0.24 ab	7.24 ± 0.06 ab	8.85 ± 0.05 a	10.13 ± 0.02 b
X_K_	107.69 ± 0.02 cde	357.53 ± 0.04 e	96.71 ± 2.14 de	132.04 ± 4.30 e	160.41 ± 0.01 de	135.96 ± 0.02 cde	384.35 ± 0.03 e	112.71 ± 0.04 de
X_OM_	5.00 ± 0.01 bcde	6.52 ± 0.01 bcd	5.40 ± 0.01 b	10.02 ± 0.06 de	6.38 ± 0.03 cde	4.09 ± 0.01 bcde	7.22 ± 0.03 bcd	6.50 ± 0.01 b
X_TN_	0.23 ± 0.02 e	0.25 ± 0.001 bc	0.20 ± 0.004 e	0.29 ± 0.005 de	0.32 ± 0.004 e	0.17 ± 0.001 e	0.33 ± 0.001 bc	0.16 ± 0.005 e
X_TP_	0.03 ± 0.001 ab	0.11 ± 0.002 cde	0.14 ± 0.003 e	0.21 ± 0.005 bc	0.05 ± 0.001 de	0.02 ± 0.001 ab	0.15 ± 0.002 cde	0.32 ± 0.003 e
X_TK_	2.45 ± 0.01 e	1.49 ± 0.02 de	1.62 ± 0.04 de	1.77 ± 0.03 abc	2.46 ± 0.05 e	2.60 ± 0.01 e	1.56 ± 0.02 de	2.35 ± 0.04 de
X_pH_	6.20 ± 0.05 abc	7.31 ± 003 bcd	6.59 ± 0.05 cde	6.39 ± 0.07 cde	6.12 ± 0.06 abcd	6.40 ± 0.05 abc	7.09 ± 003 bcd	6.20 ± 0.05 cde
X_AMT_	7.00 ± 0.05 ab	6.90 ± 0.08 d	6.40 ± 0.04 e	4.32 ± 0.02 e	6.80 ± 0.05 cde	9.00 ± 0.05 ab	6.90 ± 0.08 d	7.60 ± 0.04 e
X_1_	−7.0 ± 0.01 bcd	−4.0 ± 0.04 abcd	−8.2 ± 0.02 de	−7.6 ± 0.05 bcd	−2.2 ± 0.04 bcd	−5.6 ± 0.01 bcd	1.5 ± 0.03 abcd	−5.0 ± 0.02 de
X_7_	19.0 ± 0.01 abc	22.5 ± 0.07 ab	23.4 ± 0.03 bcd	21.7 ± 0.04 d	15.5 ± 0.01 ab	13.4 ± 0.02 abc	16.3 ± 0.03 ab	16.8 ± 0.05 bc
X_AAT_	1847.43 ± 0.03 bc	2390.80 ± 0.05 ab	3643.87 ± 0.06 c	4108.22 ± 0.04 bcd	1265.20 ± 0.04 bcde	1526.70 ± 0.03 bc	2263.20 ± 0.05 ab	1931.52 ± 0.03 bcd
X_AHT_	40.44 ± 0.08 cd	32.80 ± 0.04 bc	39.50 ± 0.01 abc	35.00 ± 0.03 ab	31.70 ± 0.01 cd	24.60 ± 0.04 cd	30.60 ± 0.05 bc	33.90 ± 0.02 abc
X_ALT_	−24.00 ± 0.04 de	−25.50 ± 0.06 d	−26.62 ± 0.03 bcd	−24.24 ± 0.01 bc	−28.91 ± 0.04 abc	−23.40 ± 0.04 de	−14.4 ± 0.02 d	−20.30 ± 0.03 bcd
X_AAP_	616.32 ± 0.01 abc	635.44 ± 0.04 bc	620.30 ± 0.04 abc	530.27 ± 0.06 cde	880.30 ± 0.04 ab	627.20 ± 0.01 abc	650.45 ± 0.04 bc	642.41 ± 0.06 abc
X_ASD_	2370.12 ± 0.04 ab	2021.35 ± 0.07 bcde	1226.55 ± 0.03 bc	2659.62 ± 0.06 abc	1738.34 ± 0.03 a	2206.40 ± 0.04 ab	2020.53 ± 0.07 bcde	2246.20 ± 0.01 bc
X_FFP_	132.61 ± 15.38 abc	158.00 ± 9.66 a	230.54 ± 11.55 ab	121.52 ± 8.12 abc	180.00 ± 1.23 a	167.00 ± 15.38 abc	180.00 ± 9.66 a	147.35 ± 11.55 ab
X_ALTI_	2061.25 ± 36.55 a	2747 ± 87.32 c	2800 ± 56.72 c	2311.5 ± 23.65 b	2525.25 ± 121.66 b	3200 ± 67.54 d	3345.25 ± 210.4 d	3373.75 ± 121.41 d

Eighteen environmental factors were performed in the Table 1. For the same variable, values with no letters in common are significantly different (*P* < 0.05).

**Table 7:** *P. fruticosa* samples collected from the eight different regions.

No.	Locations	Population	Code	Coordinates	N	Altitude (m)	Soil type	Climate zones
S1	Mei county, Shaanxi	Pingansi	PAS	E107°43′N34°1′	20	2815	Dark brown soil	Semi-humid warm temperate continental monsoon climate
		Mingxingsi	MXS	E107°44′N34°0′	20	2637	Dark brown soil	Semi-humid warm temperate continental monsoon climate
		Yuhuangmiao	YHM	E107°22′N34°5′	20	1780	Dark brown soil	Semi-humid warm temperate continental monsoon climate
		Liulingou	LLG	E108°10′N33°52′	20	1013	Dark brown soil	Semi-humid warm temperate continental monsoon climate
S2	Diebu, Gansu	Zemo	ZM	E103°21′N33°45′	20	2728	Alpine meadow soil	Mountain continental climate zone
		Dalong	DL	E103°14′N35°2′	20	2620	Alpine meadow soil	Mountain continental climate zone
		Dalagou	DLG	E103°22′N33°52′	20	2677	Alpine meadow soil	Mountain continental climate zone
		Nagai	NG	E103°14′N33°51′	20	2963	Alpine meadow soil	Mountain continental climate zone
S3	Huzhu, Qinghai	Zhalongkou	ZLK	E102°34′N36°53′	20	2264	Alpine meadow soil	Semi-arid continental plateau monsoon climate zone
		Zhalonggou	ZLG	E102°37′N36°47′	20	2698	Alpine meadow soil	Semi-arid continental plateau monsoon climate zone
		Yuanlongogu	YLG	E102°27′N36°54′	20	3069	Alpine meadow soil	Semi-arid continental plateau monsoon climate zone
		Lalagou	LL	E102°42′N36°44′	20	3169	Alpine meadow soil	Semi-arid continental plateau monsoon climate zone
S4	Jingyuan, Ningxia	Baiyunshan	BYS	E106°15′N35°37′	20	2232	Gray cinnamonic soil	Humid and semi-humid temperate climate zone
		Yehegu	YHG	E106°13′N35°31′	20	2370	Gray cinnamonic soil	Humid and semi-humid temperate climate zone
		Zhiwuyuan	ZWY	E106°18′N35°22′	20	2080	Gray cinnamonic soil	Humid and semi-humid temperate climate zone
		Qiaozigou	QZG	E106°22′N35°15′	20	2564	Gray cinnamonic soil	Humid and semi-humid temperate climate zone
S5	Yongdeng, Gansu	Suoergou	SEG	E102°43′N36°40′	20	2389	Alpine meadow soil	Semi-arid continental cold temperate climate zone
		Xiahe	XH	E102°43′N36°35′	20	2733	Alpine meadow soil	Semi-arid continental cold temperate climate zone
		Dachang	DC	E102°44′N36°44′	20	2449	Alpine meadow soil	Semi-arid continental cold temperate climate zone
		Datanzigou	DTZ	E102°46′N36°33′	20	2530	Alpine meadow soil	Semi-arid continental cold temperate climate zone
S6	Shangri-la, Yunnan	Rime	RM	E99°37′N27°51′	20	3528	Subalpine shrub soil	Mountains cool temperate monsoon climate zone
		Naipi	NP	E99°36′N28°2′	20	3432	Subalpine shrub soil	Mountains cool temperate monsoon climate zone
		Xiaozhongdian	XZD	E99°56′N27°28′	20	3590	Subalpine shrub soil	Mountains cool temperate monsoon climate zone
		Mugaocun	MGC	E99°34′N27°30′	20	2250	Subalpine shrub soil	Mountains cool temperate monsoon climate zone
S7	Nyingchi, Tibet	Zhangmaicun	ZMC	E94°20′N29°40′	20	3097	Subalpine shrub soil	Temperate continental plateau climate zone
		Selong	SL	E94°11′N29°44′	20	3173	Subalpine shrub soil	Temperate continental plateau climate zone
		Pula	PL	E94°22′N29°27′	20	3256	Subalpine shrub soil	Temperate continental plateau climate zone
		Duosongba	DSB	E94°13′N29°37′	20	3855	Subalpine shrub soil	Temperate continental plateau climate zone
S8	Kangding, Sichuan	Yajaigeng	YJG	E101°57′N30°0′	20	2946	Rich in humus loam	Humid subtemperate plateau climate zone
		Laoyulin	LYL	E101°59′N29°55′	20	3788	Rich in humus loam	Humid subtemperate plateau climate zone
		Shengkangcun	SKC	E102°1′N30°4′	20	3207	Rich in humus loam	Humid subtemperate plateau climate zone
		Zhonggucun	ZGC	E101°54′N30°16′	20	3554	Rich in humus loam	Humid subtemperate plateau climate zone
