# Supplementary material for: Influence of Environmental Factors on the Active Substance Production and Antioxidant Activity in Potentilla fruticosa L. and Its Quality Assessment
Source: Sci Rep. 2016 Jul 4;6:28591. doi: 10.1038/srep28591 (PMC4931507; doi:10.1038/srep28591)
Supplement: Supplementary Information [file srep28591-s1.pdf]

Influence of Environmental Factors on the Active Substance Production and Antioxidant Activity in *Potentilla fruticosa* L. and Its Quality Assessment

Wei Liu[1†*], Dongxue Yin[2†], Na Li[3], Xiaogai Hou[1], Dongmei Wang[3], Dengwu Li[3] & Jianjun Liu[4]

[1]Agricultural College, Henan University of Science and Technology, Luoyang 471003, China, [2]College of Agricultural Engineering, Henan University of Science and Technology, Luoyang 471003, China, [3]College of Forestry, Northwest A&F University, Yangling 712100, Shaanxi, China, [4]College of Landscape Architecture and Arts, Northwest A&F University, Yangling 712100, Shaanxi, China.

Correspondence and requests for materials should be addressed to W.L. (15729111052@163.com).

[†]These authors contributed equally to this paper.

Supplementary Text S1

Detailed description/theory of statistical methods/models

Principal Component analysis (PCA)

PCA is a well-known, unsupervised pattern recognition method of data analysis. It compresses the original data, and a new set of variables called principal components (PCs) is obtained by the following equation (viz. reducing the dimension of original data)[1,2]:

$$X=TP^T$$

where X is the original data matrix, with m objects and n variables. The T and P^T are respectively, a score matrix with a new set of (m×1) vectors, pcs, and a loadings matrix with a set of (1×n) vectors, vs that is,

$$T=[pc_1, pc_2, \ldots]$$

$$P=[v_1, v_2, \ldots]$$

These pcs are linear combinations of the original variables, and are chosen to be orthogonal to each other. Each object is identified by a score value on each PC, and every variable is likewise associated with a loading on each PC. If the variable is highly correlated with this principal component, it will be given a high loading value. The loading values present minus or plus, and a loading value of zero indicates that this variable contributes no information to this principal component[3]. Both the scores and loadings together describe the PCs of the data set. In this work, the biplot was obtained for the eighteen environmental factors and it showed the inter-relationships between objects, variables, and objects and variables.

Similar researches were conducted by Peng *et al.*[2], Li *et al.*[3] and Yang *et al.*[4].

Gray correlation analysis (GCA)

Gray correlation analysis (GCA) is a system analysis technique. The result of GCA reflects the close degree of the relationship between principal behavior factors and other factors, to deduce primary factor and secondary factors[5-7]. The analysis is based on the correlation degrees of different factors. The comparison of the correlation degrees will result in the primary factor or secondary factor. Those with higher degree of correlation would exhibit stronger influence within a system[8]. Gray correlation analysis is usually applied in the indefinite system, including small sampling and poor data information system, in which limited information is available, others are not known. In the present study, relationship between the plant and environment belongs to a gray system, it is therefore suitable to be used for the analysis of the data obtained in this study.

Similar researches were conducted by Dong *et al.*[5] and Liu *et al.*[9].

Path analysis (PA)

path analysis (PA) deals with the quantitative relationship between dependent and independent variables to explain the relative significance of each factor to the dependent variables[5,10]. Two arrow lines in Figure 1 (a) between the independent variables and dependent variable represent the path where X_1-Y and X_2-Y are independent of each other. Figure 1(b) shows four arrow lines that comprise the path network where a correlation exists between X_1 and $X_{2.}$ Therefore, except for the two direct paths (X_1-Y and X_2-Y), the path network has two indirect paths attributed to $r_{12.}$ One path is generated by

the effect of X_1 on Y via X_2 (X_1-X_2-Y), and another path is made by the influence of X_2 on Y via X_1(X_2-X_1-Y). The above situation can be extended to p variables, the direct path is X_i-Y (i = 1, 2..., p), and the indirect path is X_i-X_j-Y (i, j = 1, 2…, p; $i \neq j$)[11].

Four independent variables were included in our path analysis (Figure 2). The results are shown in Table-3 in the paper. The overall effect of Xi on Y contains two parts: the direct effect of X_i on Y (b_i, X_i—Y) and the indirect effect of X_i on Y by X_j (r_{ij} b_j, X_i—X_j—Y, $i \neq j$)[12,13]. Thereby, the correlation coefficient (r_{iy}) contains the direct path coefficient (b_i, X_i—Y) and the indirect path coefficient ($r_{ij}b_j$, X_i—X_j—Y, i≠j)($r_{iy} = b_i + \sum_{j\neq1} b_j r_{ij}$). PA was conducted by statistical software SAS 9.1 (SAS Institute, Cary, NC, USA) to evaluate correlation between the active ingredients, antioxidant activity and primary environmental factors obtained by GCA. Environmental factors were used as independent variables, and active ingredients and antioxidant activity were used as dependent variables in each test.

Similar study was conducted by Yin *et al*.[14] and Liu *et al*.[9].

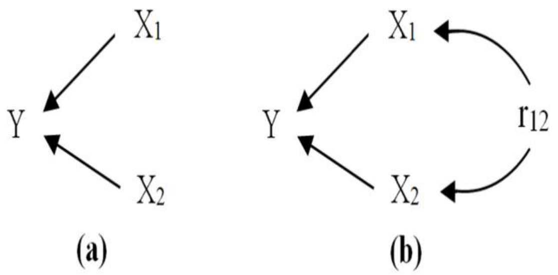

Figure-1 Path (a) and path network (b)

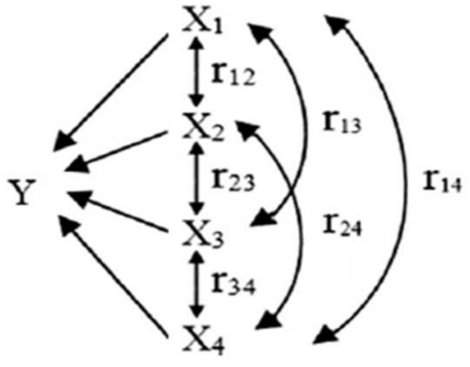

Figure-2 Network of path analysis. X_i is independent variables and Y is independent variable

Similarity analysis (SA)

HPLC fingerprint similarity analysis (SA) was performed using Computer Aided Similarity Evaluation software (CASE 2004, Zhejiang University, Hangzhou, China) as recommended by the Chinese Pharmacopoeia Committee. This software was employed to calculate the cosine values of vectorial angel among different chromatograms. The cosine value of the vectorial angle has been extensively used in evaluating similarities between different chromatograms because the method is simple, convenient and fast in the data processing[15]. The cosine values of the two chromatograms approaching 1 means they are highly similar. The cosine values can be calculated with the following

equation. This software was also used to compute the mean chromatogram as a representative standard chromatogram for a group of chromatograms. The standard HPLC fingerprint is set up with the median of all chromatograms[16–18].

$$\cos\theta = \frac{\sum_{k=1}^{m} x_{ik}x_{jk}}{\sqrt{\sum_{k=1}^{m} x_{ik}^2 \sum_{k=1}^{m} x_{jk}^2}}$$

where m is the number of common peaks; x_{ik} and x_{jk} are the relative area of peaks k of sample i and j, respectively, at the same wavelength.

Similar study was conducted by Zhou *et al.*[19].

Hierarchical clustering analysis (HCA)[2]

HCA is an on-hierarchical cluster method, i.e., clusters are not formed either by merging small groupings into larger ones or, conversely, by subdividing large clusters. Traditionally, classification implies that an object has a unique membership of a class, i.e., the objects membership of any other class is zero.

However, the HCA models attempt to assign a degree of class membership for an object over a number of classes; the method has been well described elsewhere.

The HCA method requires the user to nominate a number of classes (n) into which the data is to be divided. The data matrix is submitted to processing by a membership function, e.g.,

$$\mathrm{m(x) = 1 - c|x - a|p} \quad (\mathrm{a, c\ and\ p\ are\ constants})$$

The membership (m) values are displayed on a scale of 0 (no membership) to 1 (unique membership) with a threshold, 1/n.

An important aspect of HCA is that in addition to the above, it provides an option to hard or soft model objects. By changing the value of the index, p, on a scale of 1–3, it is possible to examine the degree of class membership (m) of any object. Thus, ideally with p = 1, an object will reside principally in its most preferred class (hard modeling), and when p = 3, the object will occupy (or spread) its membership over several classes as much as possible (soft modeling).

Thus, this technique provides an option for identifying a class of atypical objects, namely those that possess properties of more than one class. Consequently, if such objects are removed, the remaining data set will consist of objects with reasonably robust class memberships.

Similar researches were conducted by Peng *et al.*[2], Shi *et al.*[20] and Wang *et al.*[21].

Discriminant analysis (DA)

Discriminant analysis can be used to build a predictive model of the group membership based on observed characteristics of each case. This procedure generates a discriminant function (or, for more than two groups, a set of discriminant functions) based on linear combinations of the predictor variables that provide the best discrimination among the groups. The functions are generated from the samples with known membership; the functions can then be applied to new cases with measurements for the predictor variables but with unknown group membership[20]. In this study, we collected 8 samples from different areas, when we established the fingerprint, we selected 10 peaks with acceptable heights and good resolution as their common peaks,

so each case would have 10 variables. However, not all the variables are of value to the establishment of discriminant function. The procedure will generate discriminant functions only by use of valuable predictor variables. DA were performed using SPSS 19.0 (SPSS for Windows 19.0, SPSS Inc., USA)20,21. The two types of discriminant functions of *Potentilla fruticosa* generated from three different areas were as follows:

Canonical discriminant function:

$$Y_1 = 1.07X_2 - 3.351X_5 + 22.393X_8 - 1.927$$

$$Y_2 = 0.803X_2 + 2.134X_5 + 20.336X_8 - 3.733$$

Discrimination standard:

$$Y_1 > 0 \; and \; Y_1 > -Y_2: G_1$$

$$Y_1 < 0 \; and \; Y_1 < Y_2: G_2$$

$$Y_2 < 0 \; and \; Y_1 < -Y_2: G_3$$

Fisher's discrimination function:

$$G_1 = 10.853X_2 - 7.543X_5 + 192.21X_8 - 22.659$$

$$G_2 = -0.06X_2 + 25.822X_5 + 10.876X_8 - 20.512$$

$$G_3 = 2.032X_2 - 2.112X_5 + 42.323X_8 - 2.541$$

where *G1* denotes samples from the G1, *G2* denotes samples from the G2, *G3* denotes samples from the G3, and X denotes the variables. From the discriminant functions, we can see that only three variables were used to generate the functions. These three variables denoted the areas of the peaks of No. 2, No. 5 and No. 8, respectively. When we want to know which group an unknown sample is classified into, we put the values of the three variables into the three functions, respectively. The sample belongs to the group where the calculated value of the function is the highest. The result of discriminant analysis is shown in Figure-8.

Similar researches were conducted by Wang *et al*.[22], Shi *et al*.[20] and Wang *et al*.[21].

References

1. Wold, S., Esbensen K. & Geladi, P. Principal component analysis. *Chemometr. Intell. Lab.* **2**, 37–52 (1987).

2. Peng, L., Wang, Y.Z., Zhu, H.B. & Chen, Q.M. Fingerprint profile of active components for *Artemisia selengensis* Turcz by HPLC–PAD combined with chemometrics. *Food chem.* **125**, 1064-1071 (2011).

3. Li, B.Y., Hu, Y., Liang, Y.Z., Xie, P.S. & Du, Y.P. Quality evaluation of fingerprints of herbal medicine with chromatographic data. *Analytica Chimica Acta*. **514**, 69–77 (2004).

4. Yang, J., Chen, L.H., Zhang, Q., Lai, M.X. & Wang, Q. Quality assessment of *Cortex cinnamomi* by HPLC chemical fingerprint, principle component analysis and cluster analysis. *J. Sep. Sci.* **30**, 1276–1283 (2007).

5. Dong, J.E., Ma, X.H., Wei, Q., Peng, S.B. & Zhang, S.C. Effects of growing location on the contents of secondary metabolites in the leaves of four selected superior clones of *Eucommia ulmoides*. *Ind. Crop Prod.* **34**, 1607–1614 (2011).

6. Liu, G. & Yu, J. Gray correlation analysis and prediction models of living refuse generation in Shanghai city. *Waste Manage.* **27**, 345–351 (2007).

7. Yeh, Y.L. & Chen, T.C. Application of grey correlation analysis for evaluating the

artificial lake site in Pingtung Plain, Taiwan. *Can. J. Civ. Eng*. **31**, 56–64 (2004).
8. Tang, Q.Y. Date Processing System. (Science Press, Beijing, 2007).
9. Liu, W., Liu, J., Yin, D. & Zhao, X. Influence of ecological factors on the production of active substances in the anti-cancer plant *Sinopodophyllum hexandrum* (Royle) T.S. Ying. *PLoS ONE* **10**, e0122981 (2015).
10. Garson, D. Path analysis. Available at: http://ww.zozlak.org/Wnioskowanie%20Statystyczne/09_ZastosowaniaPrzyklady/Garson_2008_PathAnalysis.pdf. (Accessed: 29th June 2005)
11. Dong, M.H. Path Coefficient and Its Application. *Journal of Jiangsu Agricultural College* **4**, 45-51 (1983).
12. Garson, D. "Path Analysis" *Lecture Notes. North Carolina State University, Raleigh, North Carolina*, pp. 1-21, 2008.
13. Yuan, Z.F., Zhou, J.Y., Guo, M.C., Lei, X.Q. & Xie, X.L. Decision coefficient-the decision index of path analysis. *Journal of Northwest A&F University* **29**, 131-133 (2001).
14. Yin, D.X. *et al*. Anaerobic digestion of pig and dairy manure under photo-dark fermentation condition. *Bioresource Technol.* **166**, 373–380 (2014).
15. Fang, K.T., Liang, Y.Z., Yin, X.L., Chan, K. & Lu, G.H. Critical value determination on similarity of fingerprints. *Chemometr. Intell. Lab.* **82**, 236–240 (2006).
16. Yang, L.W. *et al*. Fingerprint quality control of Tianjihuang by high-performance liquid chromatography–photodiode array detection. *J. Chromatogr. A* **1070**, 35–42 (2005).
17. Lu, G.H. *et al*. Development of high-performance liquid chromatographic fingerprints for distinguishing Chinese Angelica from related umbelliferae herbs. *J. Chromatogr. A* **1073**, 383–341 (2005).
18. Cai, M., Zhou, Y., Gesang, S.L., Bianba, C. & Ding, L.S. Chemical fingerprint analysis of rhizomes of *Gymnadenia conopsea* by HPLC–DAD–MSn. *J. Chromatogr. B* **844**, 301–307 (2006).
19. Zhou, Y. *et al*. Chemical Fingerprinting of Medicinal Plants ''Gui-jiu'' by LC-ESI Multiple-Stage MS. *Chromatographia* **68**, 781–789 (2008).
20. Shi, X.M. *et al*. Fingerprint analysis of Lingzhi (Ganoderma) strains by high-performance liquid chromatography coupled with chemometric methods. *World J. Microbiol. Biotechnol.* **24**, 2443–2450 (2008).
21. Wang, D.M., He, F.Y., Lv, Z.J. & Li, D.W. Phytochemical composition, antioxidant activity and HPLC fingerprinting profiles of three *Pyrola* species from different regions. *PLoS ONE* **9**, e96329 (2014).
22. Wang, S., Ma, H.Q., Sun, Y.J., Qiao, C.D., Shao, S.J. & Jiang, S.X. Fingerprint quality control of *Angelica sinensis* (Oliv.) Diels by high-performance liquid chromatography coupled with discriminant analysis. *Talanta* **724**, 34–436 (2007).
